# NLRP3 inflammasome inhibitor OLT1177 suppresses joint inflammation in murine models of acute arthritis

**DOI:** 10.1186/s13075-018-1664-2

**Published:** 2018-08-03

**Authors:** Carlo Marchetti, Benjamin Swartzwelter, Marije I. Koenders, Tania Azam, Isak W. Tengesdal, Nick Powers, Dennis M. de Graaf, Charles A. Dinarello, Leo A. B. Joosten

**Affiliations:** 1 Department of Medicine, University of Colorado Denver, Aurora, CO USA; 2 Department of Rheumatology, Radboud University Medical Center, Nijmegen, The Netherlands; 3 Department of Internal Medicine and Radboud Institute of Molecular Life Sciences (RIMLS), Radboud University Medical Center, Geert Grooteplein Zuid 8, 6525 GA Nijmegen, The Netherlands

**Keywords:** IL-1β, NLRP3 inflammasome, Gout

## Abstract

**Background:**

Activation of the NLRP3 inflammasome in gout amplifies the inflammatory response and mediates further damage. In the current study, we assessed the therapeutic effect of OLT1177, an orally active NLRP3 inflammasome inhibitor that is safe in humans, in murine acute arthritis models.

**Methods:**

Zymosan or monosodium urate (MSU) crystals were injected intra-articularly (i.a.) into mouse knee joints to induce reactive or gouty arthritis. Joint swelling, articular cell infiltration, and synovial cytokines were evaluated 25 hours and 4 hours following zymosan or MSU challenge, respectively. OLT1177 was administrated intraperitoneally by oral gavage or in the food by an OLT1177-enriched diet.

**Results:**

OLT1177 reduced zymosan-induced joint swelling (*p* < 0.001), cell influx (*p* < 0.01), and synovial levels of interleukin (IL)-1β, IL-6, and chemokine (C-X-C motif) ligand 1 (CXCL1) (*p* < 0.05), respectively, when compared with vehicle-treated mice. Plasma OLT1177 levels correlated (*p* < 0.001) dose-dependently with reduction in joint inflammation. Treatment of mice with OLT1177 limited MSU crystal articular inflammation (*p* > 0.0001), which was associated with decreased synovial IL-1β, IL-6, myeloperoxidase, and CXCL1 levels (*p* < 0.01) compared with vehicle-treated mice. When administrated orally 1 hour after MSU challenge, OLT1177 reduced joint inflammation, processing of IL-1β, and synovial phosphorylated c-Jun N-terminal kinase compared with the vehicle group. Mice were fed an OLT1177-enriched diet for 3 weeks and then challenged i.a. with MSU crystals. Joint swelling, synovial IL-1β, and expression of *Nlrp3* and *Il1b* were significantly reduced in synovial tissues in mice fed an OLT1177-enriched diet when compared with the standard diet group.

**Conclusions:**

Oral OLT1177 is highly effective in ameliorating reactive as well as gouty arthritis.

**Electronic supplementary material:**

The online version of this article (10.1186/s13075-018-1664-2) contains supplementary material, which is available to authorized users.

## Background

Severe inflammation of articular joints is the hallmark of acute arthritides such as gouty arthritis and reactive arthritis. These types of acute inflammatory arthritis are initiated either by sterile triggers, such as in gout, or microbial agents, as in reactive arthritis. In the case of gout, monosodium urate (MSU) crystal deposits and a second signal are needed to trigger joint inflammation [1]. Reactive or septic arthritis is mostly caused by microorganisms such as group A *Streptococcus pyogenes*, *Chlamydia trachomatis*, *Campylobacter*, *Salmonella*, *Shigella*, or *Yersinia* [2]. Reactive arthritis occurs as a response to infections in the genitals or the gut. Of interest, these forms of acute arthritis occur mainly in males [3]; why males are more susceptible to gouty arthritis or reactive arthritis remains unknown.

A common feature of acute arthritides is the marked influx of neutrophils into the joint cavity [4]. This process is initiated mainly by the production and release of proinflammatory mediators, such as cytokines and chemokines, by the synovial lining cells. Interleukin (IL)-1β, IL-6, and IL-8 are the main mediators implicated in the onset of acute arthritis, both in humans and in murine models [5–7]. Decades ago, IL-1β was reported as the prominent cytokine for inducing production of IL-6 and IL-8 [8, 9]. IL-8 is the classical chemokine that drives large numbers of neutrophils into sites of infection or inflammation, and IL-8 has been detected in high concentrations in the synovial fluid of patients with acute arthritis [10]. In murine models of acute arthritis, IL-1β inhibition during onset of arthritis results in effective suppression of joint swelling and influx of inflammatory cells [7, 11]. These observations were further underscored by studies in which models of acute joint inflammation were induced in IL-1β-deficient mice [11].

In gout and several other forms of arthritis, nucleotide-binding oligomerization domain-like receptor (NLR) protein 3 (NACHT, LRR and PYD domains-containing protein 3 [NLRP3], cryopyrin) inflammasome activation is critical in both the inflammatory phase and the progression of disease [12]. Inflammasomes are macromolecular complexes formed in the cytosol of cells in response to various stimuli [13, 14]. Following activation of the NLRP3 inflammasome, the intracellular cysteine protease caspase-1 is activated and generates bioactive IL-1β and IL-18 [15].

We recently reported that OLT1177 (recommended International Nonproprietary Name [rINN] depansutrile) specifically targets the NLRP3 inflammasome and thereby prevents processing and release of IL-1β and IL-18 in vitro [16]. OLT1177 is active in vivo and limits the severity of endotoxin-induced inflammation [16]. The role of IL-1β in rare autoinflammatory diseases, such as familial Mediterranean fever and cryopyrin-associated periodic syndrome, is long established using specific inhibitory strategies to prevent IL-1β activity [17, 18]. Gout is a common inflammatory joint disorder, and specific inhibition of IL-1β is approved by the U.S. Food and Drug Administration and the European Medicines Evaluation Agency for reducing the number of gout attacks [19–21]. Nevertheless, a clinical need, particularly for the refractory cases, remains unmet [22]. In the current study, using two distinct murine models of acute inflammatory arthritis, we validated the rationale for treatment of acute joint inflammation with the NLRP3 inflammasome inhibitor OLT1177.

## Methods

### OLT1177 treatments

OLT1177 was synthesized as described elsewhere [16]. Crystalline OLT1177 was solubilized with sterile saline for intraperitoneal administrations and with water for oral gavage studies. OLT1177 dosing solutions were prepared fresh before each experiment.

### Mice

Animal protocols were approved by the University of Colorado Animal Care and Use Committee. Male C57BL/6 mice (10–12 weeks of age) were purchased from The Jackson Laboratory (Bar Harbor, ME, USA) and housed in the animal facility for at least 7 days before use.

### Zymosan-induced arthritis

Knee joints were injected intra-articularly (i.a.) into the synovial space directly under the patella with 180 μg of zymosan from *Saccharomyces cerevisiae* (Sigma-Aldrich, St. Louis, MO, USA). Briefly, 300 mg of zymosan were dissolved in 10 ml of sterile saline, boiled three times, and sonicated to ensure a uniform suspension. Mice were treated with OLT1177 (60, 200, or 600 mg/kg as indicated) in 200 μl of saline for intraperitoneal administration or in water for oral gavage at 24 hours, 12 hours, and 1 hour before zymosan injection. Mice received two additional administrations of the corresponding dose of OLT1177 at 11 and 23 hours after the zymosan challenge. Twenty-five hours after the zymosan instillation, mice were anesthetized, joint swelling was scored, and knee and synovial tissues were collected for histological and cytokine analyses.

### MSU crystal-induced arthritis

Gouty arthritis was induced by injecting into the right and left knees of mice 10 μl of a mixture of 300 μg of MSU crystals, 200 μM C16:0 palmitic acid (Sigma-Aldrich), and 1 mg of bovine serum albumin (Sigma-Aldrich). The effect of OLT1177 in the gouty arthritis model was tested using three different OLT1177 treatment protocols.

#### First protocol

OLT1177 and vehicle were given by oral gavage every 12 hours for five doses. One hour after the last dose, the MSU suspension was administrated in the knees of the mice.

#### Second protocol

OLT1177 and vehicle were administered orally as a single dose 1 hour after the induction of gouty arthritis.

#### Third protocol

The third protocol used a special research diet. Mice were fed either an OLT1177-enriched diet or a standard food diet for 3 weeks. The composition of the food was identical (standard mouse chow), except that OLT1177-enriched food contained 7.5 g of OLT1177 per kilogram of food. Food and water were provided ad libitum for the entire length of the study. Standard and OLT1177-enriched diets were prepared by Research Diets (New Brunswick, NJ, USA). Mice that were given i.a. injections with saline and not subjected to gouty arthritis were used as sham animals for the comparison of the joint swelling.

For each dosing protocol, mice were anesthetized, and the skin over the knee joints was opened so the MSU mixture could be injected i.a. Mice were killed 4 hours following MSU crystal instillation. The joint was exposed and scored macroscopically. Thereafter, knee joints and synovial tissue specimens were collected for histological or molecular analysis.

### Joint scoring

After the skin was removed from each knee, the joint (R and L) was scored macroscopically on a scale from 0 to 3, where 0 = no inflammation, 1 = mild inflammation, 2 = moderate inflammation, and 3 = severe inflammation, in increments of 0.25. A score of 0.25 was given when the first signs of swelling and redness were present. Joint swelling scoring was performed by two authors without knowledge of the experimental groups.

### Sample collection and cytokine measurement

After macroscopic scoring, the entire right knee joint was removed and fixed in 4% formaldehyde for histological analysis. The synovial tissue specimens from the left knee were removed for cytokine measurements. Briefly, patella with minimal surrounding muscle tissue and maximal synovial membrane was excised from the left knee joint. The synovial tissue explants were placed in 250 μl of 0.5% Triton X-100 (in water) and subjected to three freeze-thaw cycles to increase the extraction process. Cytokines were measured in the lysates of synovial tissues by specific enzyme-linked immunosorbent assays (R&D Systems, Minneapolis, MN, USA) following the manufacturer’s instructions. Because of experiment-related variations in cytokine production in some experiments, the raw data were calculated as percent changes of cytokine levels between vehicle-treated and OLT1177-treated mice. For example, in the zymosan-challenged group, each mean value in picograms per milliliter for vehicle-treated mice was set at 100%. For each value of OLT1177-treated mice, percent change was calculated. The ranges of levels in picograms per milliliter are indicated in each figure legend.

### Histological analysis

The right knee joints were fixed in 4% formaldehyde for 7 days before decalcification using 5% formic acid and processed for paraffin embedding. Tissue sections (7 μm) were stained with H&E. Histopathological changes in the knee joints were scored in the patellar/femoral region in five semiserial sections by the number of infiltrating cells in the synovial lining and/or joint cavity on a scale ranging from 0 to 3. Joint inflammation was graded on decoded slides by two separate observers.

### OLT1177 plasma exposure

OLT1177 was extracted from 50.0 μl of mouse plasma by a liquid-liquid procedure and quantified in plasma using gas chromatography (GC) with detection by MS/MS (Chemic Laboratories, Inc., Canton, MA, USA). OLT1177-D3 was used as the internal standard. Extraction started with the addition of 20.0 μl of the internal standard working solution for all samples. The samples were vortexed gently, and then 2.0 ml of ethyl acetate was added. Samples were vortexed, centrifuged, and placed in an acetone dry ice water bath, where the supernatant was transferred to clean tubes. The supernatant was then evaporated to dryness and reconstituted with *N*,O-bis(trimethylsilyl)trifluoroacetamide (1:25 vol/vol) with 1% trimethylchlorosilane/ethyl acetate. Samples were covered, vortexed, and transferred to clean amber vials. The extracts were examined by chromatograph on a DB-17 GC column (J&W Scientific, Folsom, CA, USA). OLT1177 was detected and quantified by MS/MS in positive ion mode using an Agilent 7890A GC system (Agilent Technologies, Santa Clara, CA, USA). A method qualification run was performed, and the qualified quantitation range was 20.0–2000 ng/ml.

### Whole-blood culture

Blood was collected in ethylenediaminetetraacetic acid-coated tubes, and total white blood cell (WBC) counts and the percentages of monocytes, lymphocytes, and granulocytes were determined using a HemaTrue cell counter (Heska, Loveland, CO, USA). For the whole-blood cultures, blood was diluted in RPMI 1640 medium (Mediatech CellGro; Corning, Corning, NY, USA) (1:), and 200 μl was added to each round-bottomed well. The microtiter plates were incubated for 24 hours at 37 °C. After incubation, the supernatants were removed and frozen at − 80 °C until assayed for cytokines.

### Western blotting

The protein concentration of the synovial tissue extracts (L) was determined in the clarified supernatants using a Bio-Rad protein assay (Bio-Rad Laboratories, Hercules, CA, USA). Proteins were electrophoresed on Mini-PROTEAN TGX 4–20% gels (Bio-Rad Laboratories) and transferred to nitrocellulose of 0.45 μm pore size (GE Healthcare Life Sciences, Marlborough, MA, USA). Membranes were blocked in 5% dried milk in PBS-Tween 0.5% for 1 hour at room temperature. Phosphorylated stress-activated protein kinase/c-Jun N-terminal kinase (JNK) (1:500; Cell Signaling Technology, Danvers, MA, USA), IL-1β (1:1000, AF-401; R&D Systems), and NLRP3 (1:1000, Cryo-2; AdipoGen Life Sciences, San Diego, CA, USA) were used as the primary antibodies. Peroxidase-conjugated secondary antibodies and chemiluminescence were used to develop the blots. A primary antibody against β-actin (Santa Cruz Biotechnology, Dallas, TX, USA) was used to assess protein loading.

### Statistical analysis

Statistical significance of differences was evaluated with a two-tailed Student’s *t* test using Prism version 6.0 software (GraphPad Software, La Jolla, CA, USA). Statistical significance was set at *p* < 0.05.

## Results

### OLT1177 reduces the severity of zymosan-induced arthritis

**Fig. 1:**
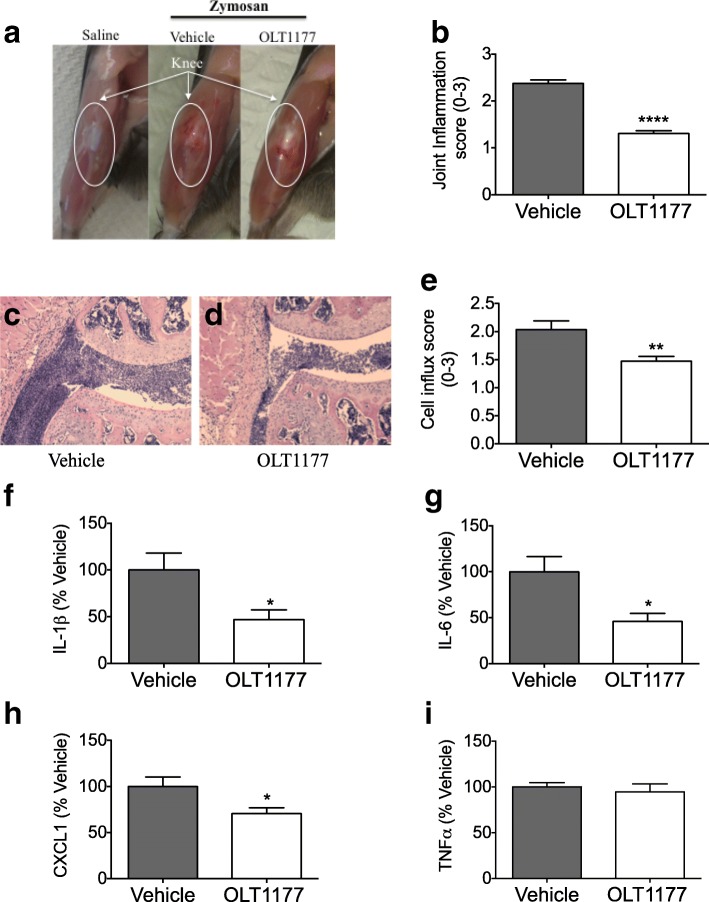
Effect of OLT1177 on zymosan-induced arthritis. **a** Representative images of knees from saline injected (Saline), vehicle-treated, and OLT1177 (200 mg/kg)-treated mice. **b** Mean ± SEM of joint score (*n* = 16 per group). **c** and **d** H&E-stained histological knee sections from vehicle- and OLT1177-treated mice. Original magnification 100 ×. **e** Mean ± SEM of cell influx (*n* = 5 per group). **f–i** Mean ± SEM percent change of interleukin (IL)-1β (range 189–2488 pg/ml), IL-6 (range 100–843 pg/ml), chemokine (C-X-C motif) ligand 1 (CXCL1) (range 169–472 pg/ml), and tumor necrosis factor (TNF)-α (282–405 pg/ml) in synovial tissue extracts from mice subjected to experimental zymosan-induced arthritis and treated with OLT1177 (200 mg/kg). Percent change was calculated as described in the Methods section of text. *n* = 8 per group; **** *p* < 0.0001, ** *p* < 0.01, * *p* < 0.05

Joint swelling and i.a. neutrophil infiltration were assessed 25 hours after injection of zymosan into the knee joints. Figure 1a depicts the marked knee joint swelling after the zymosan challenge as compared with the saline-injected group. In OLT1177-treated mice, the inflammation was reduced by 45% (*p* < 0.0001) (Fig. 1a and b). We next examined cell influx, a hallmark of inflammation and tissue damage, using histological analysis of the knee joint. H&E staining revealed that mice treated with OLT1177 had a visible reduction in infiltrating cells, predominantly neutrophils (*p* = 0.006) (Fig. 1c–e) when compared with vehicle-treated mice.

We next determined the effect of OLT1177 treatment on the levels of inflammatory mediators in inflamed synovial tissue. As depicted in Fig. 1f–h, treatment with OLT1177 (200 mg/kg) reduced synovial tissue concentrations of IL-1β and IL-6 by 55% (*p* < 0.05) and the neutrophil chemokine (C-X-C motif) ligand 1 (CXCL1) by 30% (*p* < 0.05). No significant effect was observed in tumor necrosis factor (TNF)-α levels (Fig. 1i). In addition, OLT1177 was effective in reducing joint inflammation following zymosan instillation when administrated orally. Oral gavage of OLT1177 (600 mg/kg) showed a significant reduction in synovial swelling (*p* < 0.001) and IL-1β level (− 70%; *p* = 0.01) (Additional file 1: Figure S1A and B), with no effect on TNF-α (Additional file 1: Figure S1C), when compared with vehicle-treated mice. These data indicate that OLT1177 reduced cytokine levels, which was associated with decreased severity of the zymosan-induced arthritis.

### Dose-response suppressive effect of OLT1177 on joint inflammation in mice with zymosan-induced arthritis

**Fig. 2:**
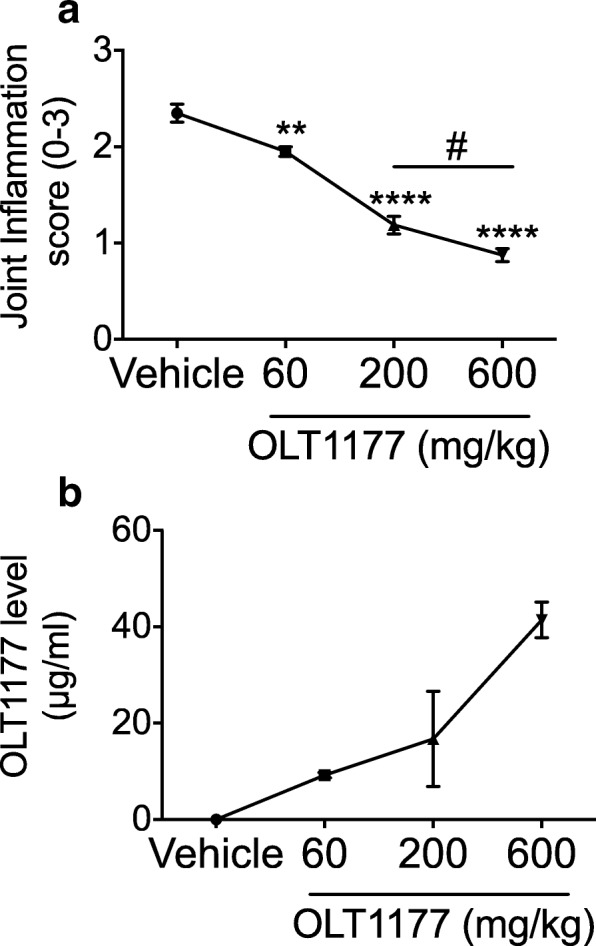
Dose-dependent effects of OLT1177 on mice subjected to zymosan-induced arthritis. **a** Mean ± SEM of joint score (*n* = 10) in mice treated with 60, 200, or 600 mg/kg of OLT1177. **b** Plasma levels (μg/ml) of OLT1177 in mice (*n* = 4–5 per group) treated with 60 (*p* < 0.01), 200 (*p* < 0.0001), or 600 mg/kg (*p* < 0.0001) of OLT1177. ^#^ 600 mg/kg vs 200 mg/kg, *** *p* < 0.001, ** *p* < 0.01, *p* < 0.05

The anti-inflammatory effect of different concentrations of OLT1177 was evaluated. As depicted in Fig. 2a, treatment with OLT1177 revealed a dose-dependent reduction of joint inflammation scores following i.a. injection of zymosan, with maximal inhibition at 600 mg/kg (− 63%; *p* < 0.0001). The different concentrations used of OLT1177 (60, 200, and 600 mg/kg) corresponded to plasma levels of 9.22 ± 0.55, 16.73 ± 9.89, and 41.4 ± 3.68 μg/ml, respectively (Fig. 2b). This analysis demonstrates a direct correlation (*p* < 0.001) between the reduction in joint inflammation and the circulating concentration of OLT1177.

### OLT1177 reduces joint inflammation of MSU crystal-induced gouty arthritis

**Fig. 3:**
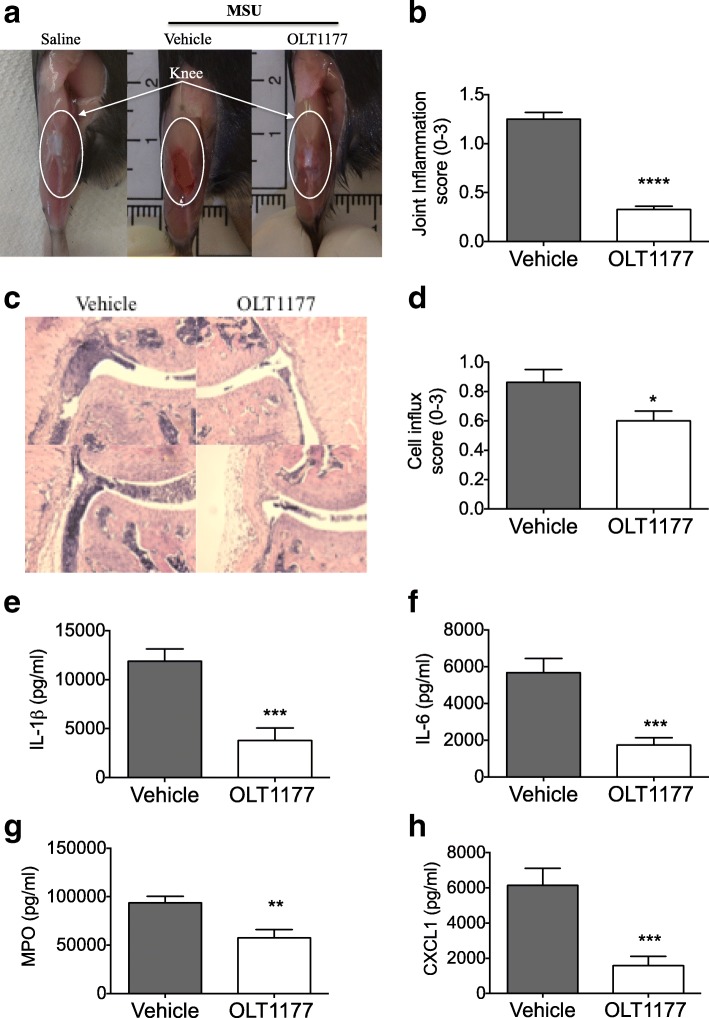
Effect of oral treatment with OLT1177 in monosodium urate (MSU)-induced arthritis. **a** Representative images of joints of saline-injected knee (Saline) and from vehicle-treated and OLT1177 (600 mg/kg)-treated mice. **b** Mean ± SEM of joint score (*n* = 20). **c** H&E-stained histological knee joint sections from vehicle- or OLT1177-treated mice. Original magnification 100 ×. **d** Mean ± SEM of cell influx (*n* = 10). **e**–**h** Mean ± SEM of interleukin (IL)-1β, IL-6, myeloperoxidase (MPO), and chemokine (C-X-C motif) ligand 1 (CXCL1) in synovial tissue lysates from mice subjected to experimental MSU-induced arthritis. *n* = 10 per group. **** *p* < 0.0001, *** *p* < 0.001, ** *p* < 0.01, * *p* < 0.05

Next, we examined the effect of OLT1177 in a model of MSU-induced gouty arthritis. Following i.a. administration of MSU crystals, severe swelling of the knee joint was observed in vehicle-treated mice (Fig. 3a). Oral treatment with OLT1177 (600 mg/kg) showed significant reduction in joint scores when compared with the vehicle group (75% reduction; *p* < 0.0001) (Fig. 3b). Histological analyses of the knee joints showed suppression of influx of inflammatory cells (31% reduction; *p* < 0.05) into the joint cavity following OLT1177 treatment (Fig. 3c, d). Synovial tissue extracts were analyzed for cytokine and chemokine concentrations. As depicted in Fig. 3e–h, there were significant reductions in IL-1β (69%; *p* = 0.004), IL-6 (70%; *p* < 0.001), myeloperoxidase (MPO) (39%; *p* = 0.006), and CXCL1 (75%; *p* < 0.001) in OLT1177-treated mice. Thus, OLT1177 reduced the local level of inflammatory mediators, which were associated with a significant amelioration in joint swelling in this model of MSU-induced gouty arthritis.

### Therapeutic administration of OLT1177 suppresses MSU-induced gouty arthritis

**Fig. 4:**
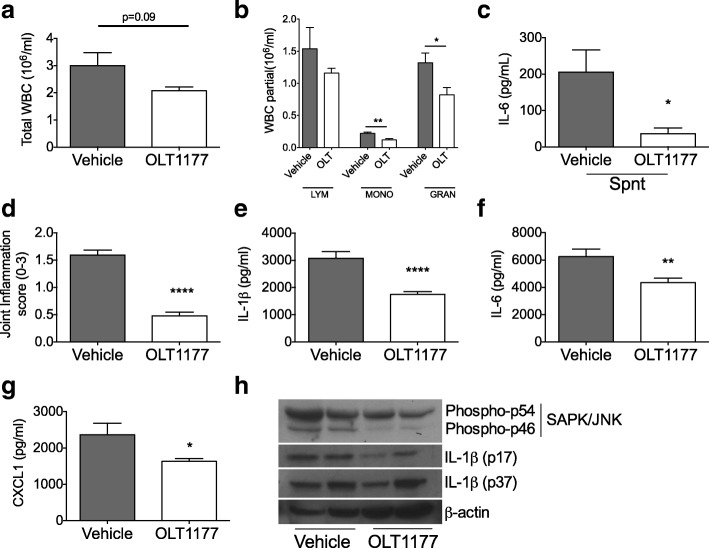
Effect of posttreatment of oral OLT1177 in monosodium urate-induced arthritis. Total white blood cell (WBC) count (**a**) and differential counts (**b**) in mice treated with vehicle or OLT1177 (600 mg/kg) (*n* = 5). **c** Mean ± SEM of spontaneous (Spnt) interleukin (IL)-6 levels from whole-blood culture in vehicle- and OLT1177-treated mice. **d** Mean ± SEM of joint score (*n* = 20). **e–g** Mean ± SEM of IL-1β, IL-6, and chemokine (C-X-C motif) ligand 1 (CXCL1) in synovial tissue extracts (*n* = 10 per group). **h** Western blot of phospho-c-Jun N-terminal kinase (JNK) and IL-1β (p17 and IL-1β precursor p37) in synovial tissue extracts. Each lane represents a single mouse. **** *p* < 0.0001, ** *p* < 0.01, * *p* < 0.05. *SAPK* Stress-activated protein kinase

Next, we evaluated the effect of oral administration of OLT1177 after the onset of arthritis to explore a therapeutic treatment strategy. Mice were injected i.a. with MSU crystals to elicit arthritis. One hour thereafter, mice received a single oral dose of OLT1177 (600 mg/kg) and were killed 3 hours later. As depicted in Fig. 4a and b, OLT1177-treated mice showed a trend toward reduced total circulating leukocytes (*p* = 0.09) and a significant reduction in monocytes (− 45%; *p* < 0.01) and granulocytes (− 38%; *p* < 0.05). Whole-blood cultures revealed that spontaneous IL-6 production by circulating WBCs was lower in OLT1177-treated mice than in the vehicle-treated group (Fig. 4c) (*p* < 0.05). Joints from mice treated with OLT1177 exhibited markedly reduced macroscopic joint inflammation (Fig. 4d) (*p* < 0.0001). In addition, in the synovial tissue extracts, IL-1β (44%; *p* = 0.0002), IL-6 (30%; *p* < 0.001), and CXCL1 (31%; *p* < 0.05) were reduced (Fig. 4e–g).

We next investigated the effect of OLT1177 treatment on the activation of the mitogen-activated protein kinase family member JNK. As depicted in Fig. 4h, phosphorylated JNK in synovial extracts from mice treated with OLT1177 was lower than in vehicle-treated mice. Reduction in IL-1β in synovial lysates was also confirmed by Western blot analysis, with reduced levels of the mature form of IL-1β (p17) in OLT1177-treated mice when compared with the vehicle-treated mice (Fig. 4h).

### OLT1177 treatment in daily food reduces MSU-induced gouty arthritis

**Fig. 5:**
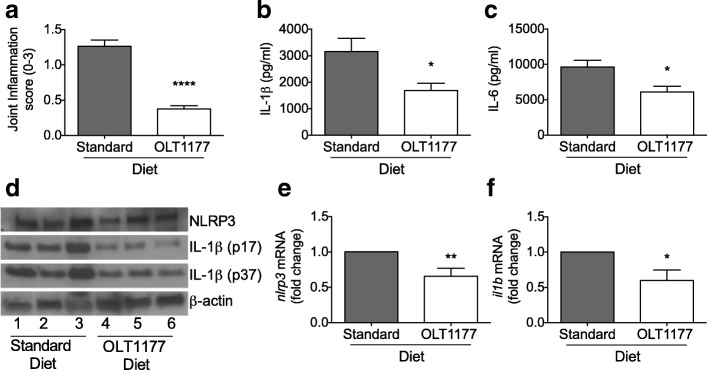
Effect of OLT1177-enriched diet in monosodium urate (MSU)-induced arthritis. **a** Mean ± SEM of joint score (*n* = 9–10). **b** and **c** Mean ± SEM of interleukin (IL)-1β and IL-6 in synovial tissue lysates from mice subjected to experimental MSU-induced arthritis (*n* = 9–10 per group). **d** Western blot for NLRP3 and IL-1β (p17 and IL-1β precursor p37) in synovial tissue lysates from mice subjected to experimental MSU-induced arthritis and fed a standard diet or an OLT1177-enriched diet. Each lane represents a single mouse. **e** and **f** Fold change of messenger RNA (mRNA) levels of *nlrp3* and *il1b* of synovial tissue extracts from mice treated with OLT1177 (*n* = 4–5 per group). **** *p* < 0.0001, ** *p* < 0.01, * *p* < 0.05

We designed a study of OLT1177 treatment to resemble an option for patients with recurrent gout attacks. Mice were fed an OLT1177-enriched diet (7.5 g of OLT1177 per kilogram of food) or a standard research diet for 3 weeks. All mice were then challenged i.a. with MSU crystals and killed after 4 hours. Figure 5a reveals significantly lower joint inflammation scores in mice fed the OLT1177 diet than in mice fed standard food. Concentrations of cytokines in the synovial tissue explants were significantly reduced for IL-1β (− 47%) and IL-6 (− 37%) (*p* < 0.05) in mice fed the OLT1177 diet compared with mice fed the standard diet (Fig. 5b, c). Both the IL-1β precursor and the active form of IL-1β (p17) were reduced in OLT1177-treated mice, as shown by Western blot analysis (Fig. 5d). No significant differences were measured in NLRP3 protein level between the two groups (Fig. 5d). Gene expression of *Nlrp3* and *Il1b* from synovial tissue extracts was reduced in the OLT1177 diet-fed mice compared with mice fed the standard diet (Fig. 5e, f).

### Comparative analysis of plasma OLT1177 concentrations following food intake in mice and in human phase I study

**Fig. 6:**
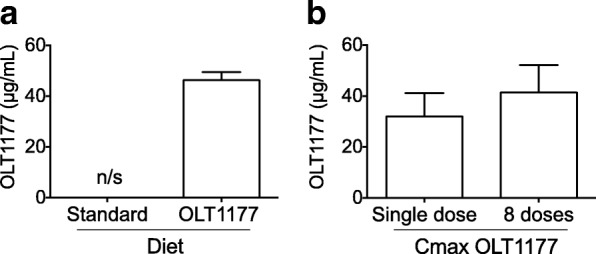
Comparison of plasma OLT1177 levels in mice and humans. **a** Mean ± SEM of OLT1177 resting plasma level (μg/ml) of mice fed for 3 weeks with standard and OLT1177-enriched diets (*n* = 5). **b** Mean ± SEM of maximum concentration (C_max_) of resting plasma levels of OLT1177 in healthy human subjects following a single dose (*n* = 5) and after eight daily oral doses (*n* = 5) of OLT1177 (16 mg/kg). Figure 6b is adapted from [16]

We next determined the concentration of OLT1177 in the plasma of mice fed the OLT1177-enriched diet for 3 weeks. During the entire duration of the study, mice had access to water and food ad libitum and were not subjected to any challenge. Figure 6a shows that mice fed the OLT1177-enriched diet reached a mean plasma OLT1177 concentration of 46.3 ± 3.15 μg/ml. Plasma OLT1177 exposure in humans was measured in a phase I clinical study in healthy subjects as described elsewhere [16]. Following a single oral dose of 1000 mg of OLT1177, the mean maximum plasma concentration (C_max_) was 32 ± 9.1 μg/ml (Fig. 6b). Plasma OLT1177 exposure in human subjects was also measured following repeated daily oral dosing for 8 consecutive days. The mean group C_max_ after eight consecutive doses of 1000 mg was 41.4 ± 10.8 μg/ml (Fig. 6b). The mean OLT1177 plasma level in mice following 3 weeks of OLT1177-enriched diet reached the same order of magnitude as the level reached in humans.

## Discussion

In the current study, we describe the anti-inflammatory effects of the synthetic small molecule sulfonyl nitrile compound OLT1177 in two different mouse models of experimental arthritis representative of reactive arthritis and gouty arthritis. As previously reported, OLT1177 in humans is safe, orally active, and specifically inhibits the NLRP3 inflammasome, preventing processing and release of active IL-1β [16]. The favorable phase I safety profile of OLT1177 combined with the reported inhibitory effects on NLRP3 inflammasome and IL-1β release led to approval of the molecule for phase II development in gout.

OLT1177 treatment administered either intraperitoneally or by oral gavage reduced joint inflammation in zymosan-induced arthritis when compared with vehicle-treated mice. We observed a significant reduction in the level of inflammatory cytokines in synovial tissue explants, including IL-1β and IL-6. Prolonged neutrophil activity, as in chronic inflammatory conditions, leads to detrimental effects [23]. In the present study, we have shown that treatment with OLT1177 suppressed cell infiltration into the joint with reduced levels of the neutrophil chemokine CXCL1 in synovial tissue extracts. These data and the reduced knee swelling in the OLT1177-treated mice are representative of the benefits of IL-1 inhibition, also observed in previous studies of reactive arthritis [7, 24].

OLT1177 is a specific NLRP3 inflammasome inhibitor [16]. Zymosan contains β-glucan from the cell wall of the yeast (*S. cerevisiae*), and this glucan induces NLRP3 inflammasome activation [25]. In the present study, we have demonstrated that OLT1177 reduces the severity of zymosan-induced arthritis in a dose-dependent manner by intraperitoneal administration and via oral gavage in mice. The lack of effect of OLT1177 treatment on TNF-α production supports previously observed data [16] confirming that OLT1177 primarily targets IL-1β.

We observed a direct correlation between the doses 60, 200, and 600 mg/kg of OLT1177 and the reduction in joint inflammation (− 17, *p* < 0.01; − 50, *p* < 0.0001; and − 63%, *p* < 0.0001, respectively). In comparison, human exposure after oral administration of OLT1177 led to an average C_max_ of 41.4 ± 10.8 μg/ml after 1000 mg/d for 8 consecutive days [16], which is of the same order of magnitude achieved in the 600 mg/kg dose group. Thus, the OLT1177 exposure used in this study, which was shown to be effective in reducing the severity of zymosan-induced arthritis in a mouse model, reached plasma levels similar to those observed in humans, with no significant adverse effects [16].

Gouty arthritis is a specific form of inflammatory arthritis with elevated urate levels in the bloodstream [3, 26]. In this condition, formation and deposition of MSU crystals in the synovial space cause acute inflammation due to neutrophil infiltration. Acute gout manifestations include attacks of severe pain, stiffness, and swelling of a distal joint, with great impact on patient quality of life [27]. In the present study, we have demonstrated that oral treatment with OLT1177 reduced joint swelling and infiltration of inflammatory cells in a murine model of gouty arthritis simulated by administration of MSU crystals into the articular space. Compared with the vehicle-treated group, mice treated with OLT1177 exhibited significant reductions in IL-1β, IL-6, MPO, and CXCL1 levels in extracted synovial membranes. These data support the therapeutic potential of the use of OLT1177 in gout, an IL-1β-mediated disease [4, 28].

In this study, treatment with OLT1177 was consistently associated with a reduction in IL-6. Although the properties of IL-6 during an acute-phase response are well known, the role of IL-6 in gout remains unclear. Mokuda et al. showed that treatment with tocilizumab improved clinical symptoms in a patient with systemic tophaceous gout [29]. Pinto et al. showed the benefit of tocilizumab treatment in another case of gouty arthritis not responding to the nonsteroidal anti-inflammatory drugs colchicine and allopurinol [30]. However, unlike IL-1β blockade, there are no randomized clinical trials of tocilizumab in refractory gout. It is likely that the increase in IL-6 in gout is a biomarker for active IL-1β. In fact, the ability of IL-1β to induce IL-6 is long established, and elevated IL-6 is often used as a surrogate marker for subpicogram levels of IL-1β in humans. In mice, IL-6 is decreased by genetic and pharmacologic inhibition of IL-1 in gouty arthritis [31]. Further, IL-6 functions as a marker in inflammasome-mediated inflammation with no direct role in MSU-induced inflammation [32]. The acute inflammation of arthritis induced by zymosan is IL-6-independent [33]. In the present study, the reduction in IL-6 is likely due to OLT1177-mediated IL-1β reduction as a consequence of the NLRP3 inflammasome inhibition.

We also evaluated the anti-inflammatory effect of therapeutically administrated OLT1177. To this end, we designed a study that mimics a clinical setting where a subject begins treatment after the onset of clinical disease. Mice were subjected to an i.a. injection of MSU crystals, and after 1 hour, vehicle or OLT1177 was administrated as a single oral dose. Mice were killed 3 hours following treatment. A single dose of OLT1177 given therapeutically reduced joint inflammation as well as IL-1β, IL-6, and CXCL1 concentrations in the extracted synovial tissue. The anti-inflammatory properties of OLT1177 treatment in the MSU-induced arthritis model were also confirmed with the reduction in the phosphorylation of JNK, which has been implicated in the pathophysiology of several forms of arthritis, such as rheumatoid arthritis and gouty arthritis [34–36].

Patients with recurrent attacks of gout have been treated chronically with IL-1β-blocking therapies (anakinra [37, 38], canakinumab [39, 40], or rilonacept [41, 42]) and have significantly reduced attack rates. Considering the safety profile of oral OLT1177 in humans [16], we designed a study of prolonged oral OLT1177 exposure. Mice were fed a standard diet or an OLT1177-enriched diet for 3 weeks before i.a. injection of MSU crystals into the joints. Joint inflammation and inflammatory markers were reduced in the mice fed an OLT1177 diet when compared with the standard diet group. In addition, Western blot analysis of the synovial membranes revealed a reduction in IL-1β precursor (p37) as well as the mature form of IL-1β (p17) with no change in NLRP3 protein level. These data are consistent with the autopositive feedback of IL-1, where IL-1 induces IL-1 [43]. Thus, we postulate that chronic OLT1177-mediated suppression of active IL-1β production interrupted IL-1β-induced IL-1β precursor synthesis and not solely the NLRP3-dependent release of bioactive IL-1β and the downstream effect of IL-1β.

The unchanged NLRP3 protein level between the two treatment groups indicates that OLT1177 does not reduce inflammasome protein levels in the short-term model (4 hours). We previously reported that there is no reduction in NLRP3 in cells treated in vitro with OLT1177 [16]. However, we did observe reduced *Nlrp3* gene expression in the OLT1177-enriched diet group compared with the standard diet mice. Because messenger RNA was extracted from whole synovial tissue, the reduction in *Nlrp3* gene expression likely reflects a reduction of cell influx into the synovial membrane in the OLT1177-treated mice. Alternatively, OLT1177 in the food may have reduced gene expression of *Nlrp3* during the 3 weeks of the enriched diet. Mean plasma levels of OLT1177 after the 3 weeks was 46.3 ± 3.15 μg/ml (347 μM). In humans, after 8 days of oral OLT1177, the mean plasma level was 41.4 μg/ml (311 μM). In vitro, the half maximal inhibitory concentration of OLT1177 for IL-1β secretion was 1 μM for human blood monocyte-derived macrophages [16]. Thus, on the basis of efficacy in vitro, OLT1177 reaches plasma levels 300-fold greater than those needed to reduce IL-1β secretion in primary human cells.

## Conclusions

There is a growing body of research substantiating the mechanism and efficacy of NLRP3 inflammasome inhibitors in experimental animal models, including acute arthritides. However, no approved human agents are available. The small and orally active molecule OLT1777 inhibits the formation of the NLRP3 inflammasome and reduces the severity of reactive and gouty arthritis. On the basis of evidence described in this study and the favorable phase I safety profile, oral OLT1177 has advanced into three phase II development programs, including gout, establishing the translational value of OLT1177 as a safe oral NLRP3 inhibitor.

## Additional file

Additional file 1: **Figure S1.** Effect of oral treatment with OLT1177 in zymosan-induced arthritis. **a** Mean ± SEM of joint score (*n* = 8). **b** and **c** Mean ± SEM of IL-1β and TNF-α in synovial tissue extracts from mice subjected to experimental zymosan-induced arthritis and treated with OLT1177 (600 mg/kg) (*n* = 4 per group). **** *p* < 0.0001, * *p* < 0.05 vs vehicle. (DOCX 47 kb)

## Abbreviations

C_max_: Maximum plasma concentration
CXCL1: Chemokine (C-X-C motif) ligand 1
GC: Gas chromatography
i.a.: Intra-articularly
IL-18: Interleukin 18
IL-1β: Interleukin 1β
IL-6: Interleukin 6
IL-8: Interleukin 8
JNK: c-Jun N-terminal kinase
MPO: Myeloperoxidase
mRNA: Messenger RNA
MSU: Monosodium urate
NLR: Nucleotide-binding oligomerization domain-like receptor
NLRP3: NACHT, LRR and PYD domains-containing protein 3
rINN: Recommended International Nonproprietary Name
SAPK: Stress-activated protein kinase
TNF-α: Tumor necrosis factor-α
WBC: White blood cell
