# Supplementary material for: NLRP3 inflammasome inhibitor OLT1177 suppresses joint inflammation in murine models of acute arthritis
Source: Arthritis Res Ther. 2018 Aug 3;20:169. doi: 10.1186/s13075-018-1664-2 (PMC6091035; doi:10.1186/s13075-018-1664-2)
Supplement: Supplementary file 1 — Figure S1. Effect of oral treatment with OLT1177 in zymosan-induced arthritis. a Mean ± SEM of joint score (n = 8). b and c Mean ± SEM of IL-1β and TNF-α in synovial tissue extracts from mice subjected to experimental zymosan-induced arthritis and treated with OLT1177 (600 mg/kg) (n = 4 per group). **** p < 0.0001, * p < 0.05 vs vehicle. (DOCX 47 kb) [file 13075_2018_1664_MOESM1_ESM.docx]

**Additional file 1**

**Figure S1. Effect of oral treatment with OLT1177 in zymosan-induced arthritis.** (A) Mean ±SEM of joint score (N=8). (B, C) Mean ±SEM of IL-1β and TNFα in synovial tissue extracts from mice subjected to experimental zymosan-induced arthritis and treated with OLT1177 (600 mg/kg). N=4 per group. ****: p<0.0001, *: p<0.05 vs vehicle.
